# Genome-Wide Demographic Analyses of Balaenid Whales Revealed Complex History of Gene Flow Associated with Past Climate Oscillation

**DOI:** 10.1093/gbe/evaf081

**Published:** 2025-05-05

**Authors:** Bai-Wei Lo, Francisca Martinez Real, Andreas Magg, John Pierce Wise, Stefan Mundlos, Paolo Franchini

**Affiliations:** Research Group of Development and Disease, Max Planck Institute for Molecular Genetics, Berlin, Germany; Research Group of Development and Disease, Max Planck Institute for Molecular Genetics, Berlin, Germany; Andalusian Center for Developmental Biology, CABD (UPO-CSIC-JA), Seville, Spain; Research Group of Development and Disease, Max Planck Institute for Molecular Genetics, Berlin, Germany; BCRT, Berlin Institute of Health (BIH), Charité Universitätsmedizin, Berlin, Germany; Wise Laboratory of Environmental and Genetic Toxicology, Department of Pharmacology and Toxicology, University of Louisville, Louisville, KY, USA; Research Group of Development and Disease, Max Planck Institute for Molecular Genetics, Berlin, Germany; Institute of Medical and Human Genetics, Charité Universitätsmedizin, Berlin, Germany; Department of Ecological and Biological Sciences, University of Tuscia, Viale dell'Università s.n.c, Viterbo, Italy

**Keywords:** gene flow, introgression, Balaenidae, right whale, bowhead whale, cetaceans

## Abstract

The balaenid whale, comprising three species of right whales and the bowhead whale, represents an ancient and highly endangered lineage of marine mammals. To unravel the evolutionary history of balaenid whales with respect to gene flow, a comprehensive analysis based on whole-genome data was conducted for all species within this group. Employing population genomic methodologies, we revealed that extant right whales form an unresolved branching pattern, identified evidence of historical transequatorial migration, and provided estimates of the age of the group. Furthermore, we investigated the impact of glacial cycles on the connectivity of bowhead whale populations. By employing multiple complementary approaches to detect gene flow, we identified and characterized gene flow events from bowhead whales to North Atlantic right whales, offering detailed insights into the process. Lastly, we assessed the phenotypic consequences of interspecies gene flow. Our study sheds light on the intricate evolutionary history of modern balaenid whales, which have been profoundly shaped by ancient climate events.

**Significance**

While interspecific gene flow is known to be widespread among animals, including cetaceans, the driver, and impact of these genetic exchanges, especially within the ancient and endangered balaenid whales (bowhead whale and the right whales), remain poorly understood. This study reveals significant waves of gene flow among major lineages in the balaenid whales. These findings enhance our understanding of the evolutionary dynamics and historical gene flow in the balaenid whales, highlighting the profound effects of ancient climate events on their genetic diversity.

## Introduction

Interspecific introgression, the incorporation of genetic material from one species into the gene pool of another through repeated backcrossing (Rieseberg and Carney 1998), has long been considered infrequent. However, it is now recognized as widespread among diverse taxonomic groups (Edelman and Mallet 2021; Dagilis et al. 2022). The increased availability of genomic data from nonmodel organisms, coupled with recent advancements in analytical methodologies, has not only facilitated the identification of interspecific introgression across different taxa but also significantly enhanced our ability to characterize the introgressed genetic material. This, in turn, allows for a more comprehensive assessment of the evolutionary consequences associated with this process (Hibbins and Hahn 2022). Nevertheless, the evolutionary significance of interspecific introgression and its impact on local adaptation and speciation remain active research areas (Edelman and Mallet 2021; Fontaine 2022).

In cetaceans, interspecific introgression has been documented in both baleen and toothed whales, with several studies highlighting ancient introgression events during the early stages of diversification (Árnason et al. 2018; Moura et al. 2020; Guo et al. 2021; Westbury et al. 2023). These introgression events are often the results of extensive gene flow, defined here as movement of genetic material between populations of the same or different species. This process in cetaceans was likely facilitated by the absence of significant extrinsic barriers in the marine environment. However, evaluating the driving forces and evolutionary implications of these ancient events remains challenging. Most studies have focused on historical introgression without fully addressing how contemporary processes, such as ongoing gene flow or adaptation to climate change, contribute to the evolutionary history of cetaceans.

The family Balaenidae presents a unique opportunity to further investigate interspecific introgression while addressing existing challenges. Balaenidae is the sibling group to Plicogulae, which includes rorquals, the gray whale, and the pygmy right whale. Together, these groups comprise modern baleen whales. There are only four extant balaenid species: the bowhead whale (*Balaena mysticetus*) and three species of right whales—the North Pacific right whale (*Eubalaena japonica*), the southern right whale (*Eubalaena australis*) and the North Atlantic right whale (*Eubalaena glacialis*), which is currently one of the most endangered large whale species (IUCN, 2023). Balaenid whales inhabit temperate (right whales) to Arctic (bowhead whale) waters in both hemispheres. The recent divergence of modern balaenid whale species provides a unique opportunity to explore the gene flow that occurred around the time of speciation. Furthermore, the cold-adapted nature of modern balaenid whales has likely made them more sensitive to global climate events than other cetaceans, enabling a more thorough assessment of the evolutionary significance of introgression.

In this study, we present the first genome-wide demographic analysis of all four balaenid whale species. By employing complementary genomic approaches, we reconstructed a detailed picture of their divergence and gene flow dynamics. We shed light on the phylogenetic relationships among modern right whales, estimated the age of each taxonomic group, described different scenarios of intra and interspecific gene flow, and inferred the phenotypic consequences of interspecific gene flow. Our findings provide novel insights into the evolutionary history of this ancient surviving whale lineage and highlight the role of ancient climate events in shaping patterns of introgression. This research enhances our understanding of the complex dynamics of gene flow and divergence in this group of marine mammals.

## Results

### Phylogenomic Analyses Reveal Conflicting Phylogenetic Signals in Modern-Day Right Whales

**Fig. 1.**
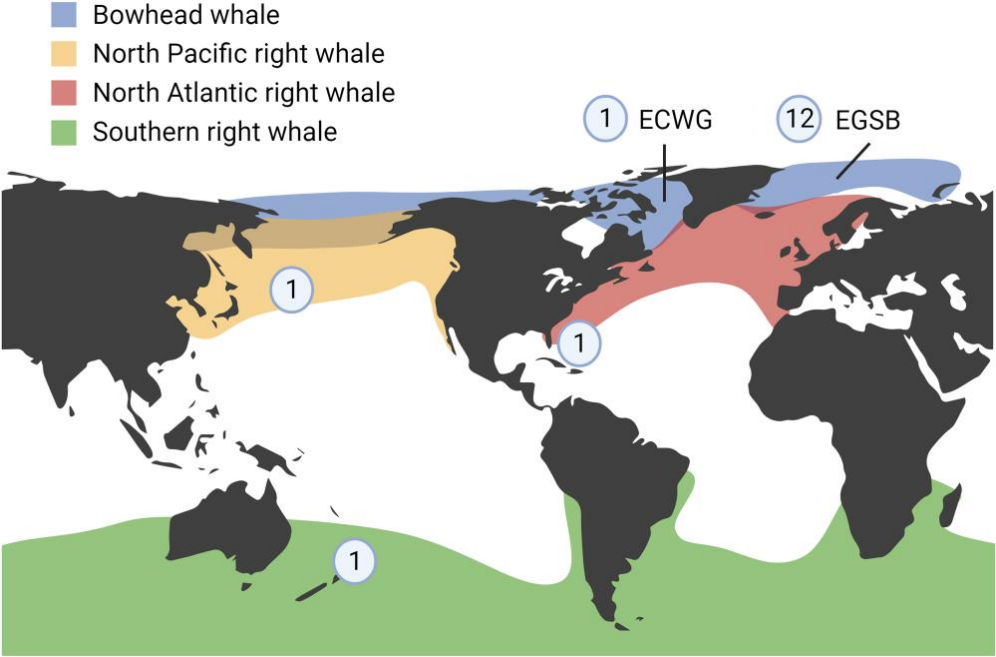
Balaenid whale samples used in the study. Geographical distribution of the four balaenid whale species: the bowhead whale (*B. mysticetus*), the North Pacific right whale (*E. japonica*), the southern right whale (*E. australis*), and the North Atlantic right whale (*E. glacialis*). Numbers indicate the sample size for each species, including individuals from the ECWG and EGSB bowhead whale populations. The distribution data were adapted from Tsai and Chang (2019) and George and Thewissen (2020). Created with BioRender.com.

**Fig. 2.**
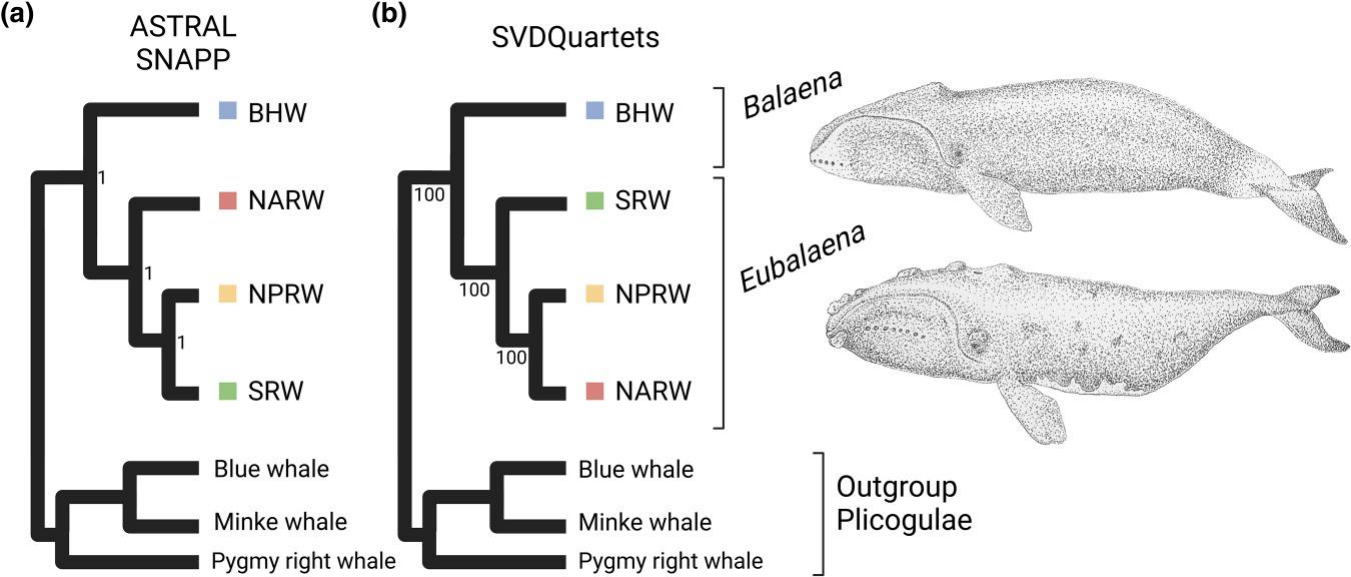
Phylogenetic relationships of balaenid whales inferred from whole genomic data. a) As inferred from 50 kbp genomic windows, ASTRAL and SNAPP results show that the North Atlantic right whale is the sister group to the clade that includes the North Pacific and the southern right whales within the *Eubalaena* genus. b) SVDQuartets results, inferred from genome-wide SNVs, suggest a different relationship, where the southern right whale is the sister group to the other two *Eubalaena species*, which form a monophyletic northern group. All branches were supported by 100% posterior probabilities (ASTRAL and SNAPP) or 100% bootstrap values (SVDQuartets). The discrepancy of the results highlights the conflicting phylogenetic signals in the evolutionary history of right whales. Balaenid whales illustrated by B.-W.L. Created with BioRender.com.

To understand the evolutionary relationships of balaenid whales, we collected cetacean genomic data from public databases (Fig. 1; supplementary table S1, Supplementary Material online). We performed phylogenomic analyses on the four focal balaenid species while using three Plicogulae species as outgroups: minke whale (*Balaenoptera acutorostrata*), blue whale (*Balaenoptera musculus*), and pygmy right whale (*Caperea marginata*). We estimated species trees independently using three multispecies coalescent model-based programs. In brief, multispecies coalescent models assume there exists a true dichotomous species tree and infer this species tree by modeling the process of incomplete lineage sorting. First, we used ASTRAL, a summary statistic-based model that infers a species tree from a collection of subtrees (Zhang et al. 2018). We cut the genomes into 28,586 nonoverlapping fragments that were 50 kbp in length and used RAXML to estimate the topology of each subtree. We forwarded the topologies of the subtrees to ASTRAL to estimate the species tree. The result of ASTRAL showed a sibling relationship between North Pacific right whale and southern right whale, with all branches having 100% posterior probability support (Fig. 2). Second, we applied the multispecies coalescent-based models (MCMCs) implemented in SVDQuartets and SNAPP, both of which infer species trees from genome-wide single nucleotide variants (SNVs). Interestingly, the results of SNAPP agreed with those of ASTRAL, whereas SVDQuartets inferred a completely different relationship among the right whales. The tree inferred by SVDQuartets revealed a monophyletic northern group comprising the two northern right whale species, with each node supported by a 100% bootstrap value (Fig. 2). The inconsistency in reconstructing species trees, even when using whole-genome data, underscores the challenge of accurately depicting the evolutionary history of balaenid whales through traditional dichotomous phylogeny.

### Phylogenetic Network and *D*-statistics Show Interspecies Gene Flow among Balaenid Whales and between Major Baleen Whale Lineages

**Fig. 3.**
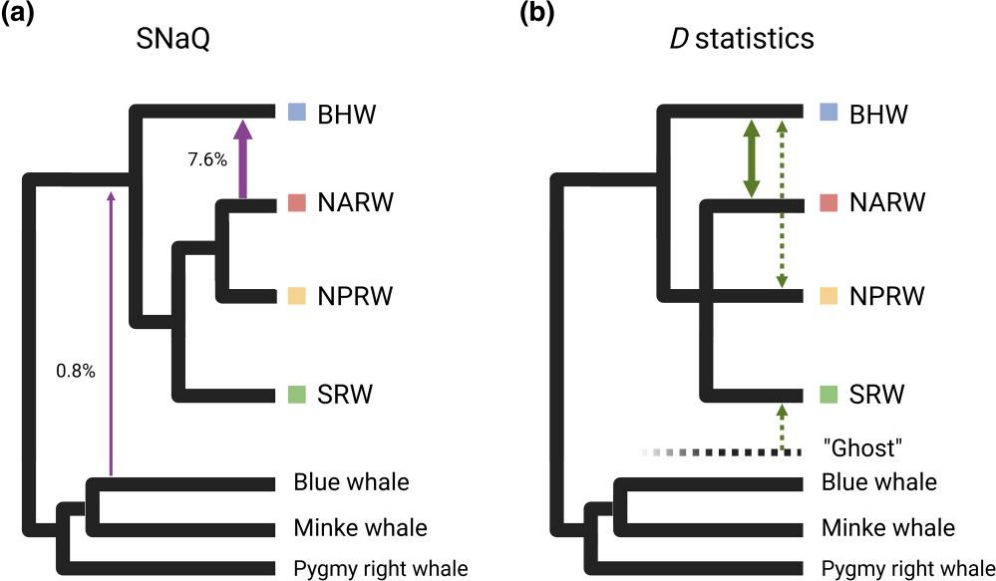
Interspecific introgression inferred from SNaQ and *D*-statistics. a) Phylogenetic network analysis carried out with SNaQ revealed two waves of gene flow (arrows), with introgression levels of 7.6% from the North Atlantic right whale to the bowhead whale and 0.8% from the blue whale to the common ancestors of balaenid whales. b) *D*-statistics again discovered introgression between the bowhead whale and North Atlantic right whale (solid arrow), with potential gene flow events (dashed arrows) explaining excessive allele sharing between the bowhead whale and North Pacific right whale. These events could involve direct gene flow or gene flow from a “ghost” lineage, whose position ranges from being the sister group to the balaenid clade to being within the outgroups. Both analyses support interspecific gene flow among balaenid species. Created with BioRender.com.

One potential explanation for the discordant topologies obtained with different phylogenomic methods used to resolve the relationship of balaenid whales is the presence of interspecific gene flow. To take gene flow into account, we estimated phylogenetic networks from genome-wide SNVs, again using the three Plicogulae species as outgroups. Phylogenetic networks have the advantage of simultaneously estimating species tree and gene flow events. To this end, we used the maximum pseudolikelihood algorithm implemented in SNaQ. Because SNaQ can only infer level one networks in order to avoid overfitting, the maximum number of gene flow events that can be predicted in our seven species setting is two. Like SVDQuartets, SNaQ also inferred a sibling relationship of two northern right whales, with 100% bootstrap support for this clade. The outputs of SNaQ are unrooted networks and have no branch lengths, thus allowing gene flow estimation between Balaenidae and Plicogulae. The best scored phylogenetic network contains two waves of gene flow: (i) from the North Atlantic right whale to the bowhead whale (7.6% introgression) and (ii) from the blue whale to the common ancestors of balaenid whales (0.8% introgression) (Fig. 3a). The inferred network is able to better explain gene tree discordance than the dichotomous tree (supplementary fig. S1, Supplementary Material online). The result from the phylogenetic network indicates the presence of interspecific gene flow.

To systemically survey for signals of gene flow, we applied genome-wide *D*-statistics in selected trios of balaenid species with Dsuite. *D*-statistics is a nonparametric method for detecting gene flow (Patterson et al. 2012). It takes three taxa and an outgroup and tests for excessive allele sharing between the nonsister taxa. Excessive allele sharing between taxa can indicate gene flow. Since the *D*-statistics require a known dichotomous relationship of the tested taxa, we therefore only applied it on the bowhead whale plus a pair of right whales, using the three Plicogulae species as outgroups. We found significant results in all three tested trios (Table 1). Excessive allele sharing was found between the bowhead whale and North Atlantic right whale and between the bowhead whale and North Pacific right whale. The level of sharing was most prominent between the bowhead whale and North Atlantic right whale (Table 1). This high level of allele sharing between the bowhead whale and North Atlantic right whale strongly supports gene flow between the two (Fig. 3b). In contrast, the lesser signal of excessive allele sharing between the bowhead whale and North Pacific right whale can be explained by direct gene flow between bowhead whale and North Pacific right whale, or nonmutually exclusively by gene flow from an unsampled lineage to southern right whale (Fig. 3b). To explore whether the sex chromosome is also affected by introgression, we estimated the *D*-statistics on the X chromosome. Surprisingly, the X chromosome harbors more introgression in the bowhead whale—North Atlantic right whale introgression than autosomes (*D* value 0.154 vs. 0.114).

**Table 1:** Results of *D-*statistics

P1	P2	P3	D	*P* value	*Z* score
NPRW	NARW	BHW	0.113	<0.001	49.3
SRW	NARW	BHW	0.275	<0.001	89.3
SRW	NPRW	BHW	0.173	<0.001	75.1
EGSB BHW	ECWG BHW	NARW	0.297	<0.001	64.8
PRW	BW	SRW	0.03	<0.001	22.9

Note that the tree provided to *D*-statistics is (((P1, P2), P3), Outgroup). NPRW: North Pacific right whale; NARW: North Atlantic right whale; BHW: bowhead whale; SRW: southern right whale; BW: blue whale; PRW: pygmy right whale.

To provide further understanding on the blue whale—balaenid whale introgression inferred from phylogenetic network, we applied *D*-statistics on southern right whale, pygmy right whale, and blue whale, using sperm whale (*Physeter macrocephalus*) as an outgroup. We detected significant excessive allele sharing between southern right whale and blue whale (Table 1), which supports the result of phylogenetic network. In summary, our genome-wide surveys from both phylogenetic network and the *D*-statistic painted a similar picture of interspecific gene flow among balaenid whales. Both analyses support (i) North Atlantic right whale—bowhead whale and (ii) blue whale—balaenid whale introgressions. *D*-statistics also suggests the potential occurrence of gene flow between North Pacific right whale and bowhead whale and/or southern right whale and an unsampled lineage (ghost).

### Haplotype-Based Method MSMC-IM Confirms the Increased Connectivity of Atlantic Bowhead Whale Populations during Glacial Periods

**Fig. 4.**
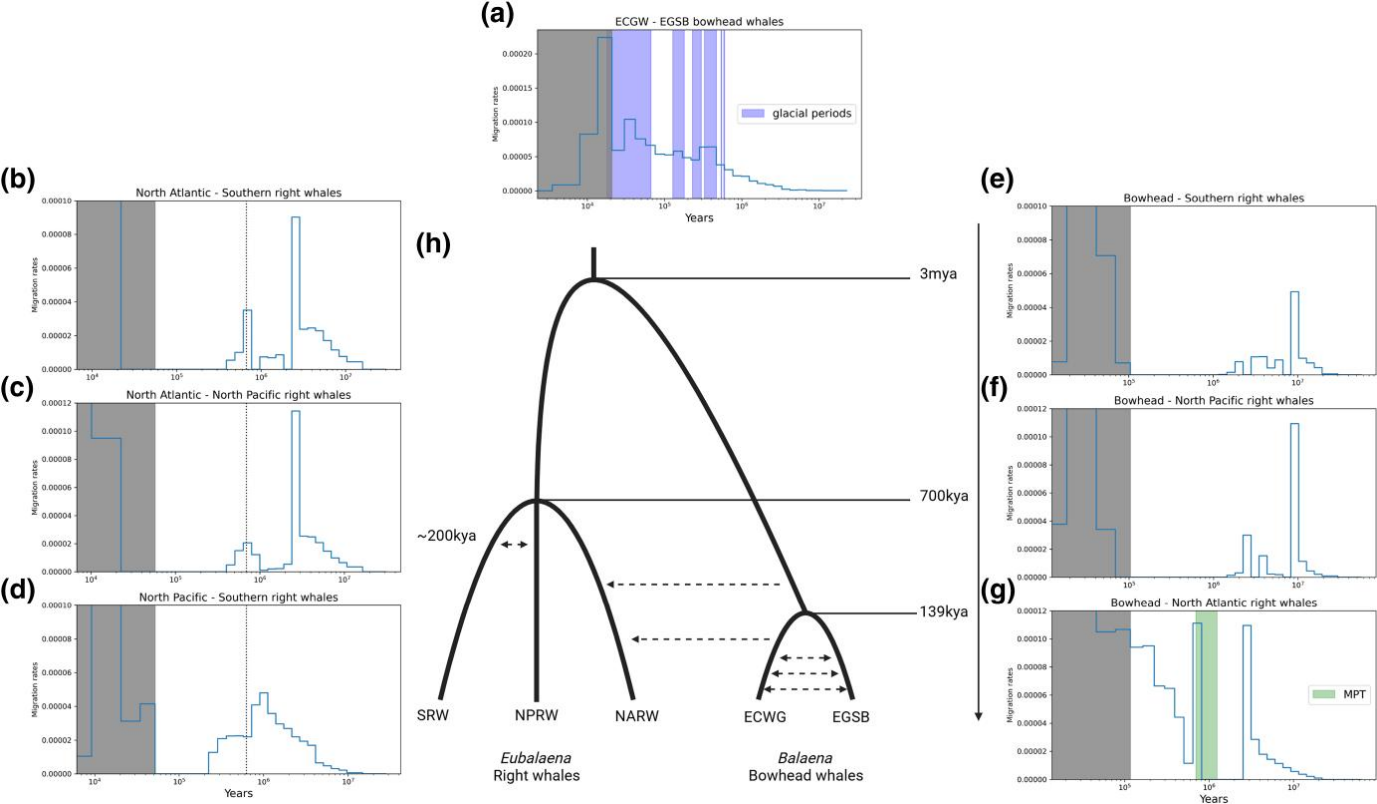
Intra- and interspecific migration profiles estimated from MSMC-IM. a) Increases in migration rate between two populations of bowhead whales (ECWG and EGSB) align with Pleistocene glacial periods (highlighted bars), consistent with predicted southward movement during the Last Glacial Maximum. b and c) Signals of archaic introgression are evident in species pairs involving the North Atlantic right whale, with peaks occurring approximately 2 to 3 million years ago. These introgression peaks suggest contributions from an unsampled divergent lineage predating the main divergence. Dashed lines represent the 50% cumulative migration rate, indicating the point of major divergence between species pairs. d) Continuous gene flow between the North Pacific right whale and southern right whale persisted until approximately 200,000 years ago, revealing evidence of late transequatorial migration through the Pacific Ocean. e and f) Minimal postdivergence gene flow is detected between bowhead whales and either the North Pacific or southern right whales. g) Introgression from the ECWG bowhead whale population to the North Atlantic right whale began during the mid-Pleistocene transition (highlighted area) and persisted until the recent past. Gray-shaded areas indicate manually masked regions with potential phasing artifacts. h) Phylogenetic representation showing divergence time inferred from pairwise 50% cumulative migration rate. Arrows represent introgressions and their directions inferred with pairwise MSMC-IM. Created with BioRender.com.

We discovered signals of introgression among balaenid whales, which contradicts basic assumptions of traditional phylogenetic analyses. Still, the evolutionary history of modern right whales remains unresolved. Further details regarding the introgression events also need to be elaborated. These details include timing and direction of gene flow and whether the gene flow came from secondary contact or isolation with migration. In order to address these questions, we turned to MSMC-IM, a haplotype-based population genomic approach for demographic analysis. MSMC-IM infers a time-dependent gene flow model between pairs of taxa. MSCM-IM can also detect introgression signals from unsampled divergent lineages, also known as archaic introgression. To demonstrate the power of this haplotype-based approach on inferring migration history, we first used MSMC-IM to reconstruct the intraspecific migration within bowhead whales in the North Atlantic Ocean. The East Greenland-Svalbard-Barents Sea (EGSB) and East Canada-West Greenland (ECWG) populations are currently predominantly separated by Greenland (Fig. 1), but were predicted to come into contact when they were forced south during the Last Glacial Maximum (Foote et al. 2013). Indeed, we detected periodic changes in migration rate between the two populations using MSMC-IM (Fig. 4a). Previous estimations for the generation time of bowhead whales varied quite differently (35 vs. 52 years) (Rooney et al. 2001; Taylor et al. 2007), and we therefore use the glacial cycles and MSMC-IM result to evaluate which of these two estimates is more accurate. The rises in migration rate aligned almost perfectly with Pleistocene glacial periods when the generation time is set to 35 (Fig. 4a; supplementary fig. S2, Supplementary Material online). The divergence time estimated for the two populations is 139 Kya (Fig. 4h; supplementary fig. S3, Supplementary Material online). Our finding provides direct molecular evidence on the influence of glacial cycles on migration in bowhead whales, while also bringing insights into the life history of this species.

### MSMC-IM Reveals Overlapping Divergence Times among Extant Right Whales and Evidence of Transequatorial Migration through the Pacific Ocean

To understand the detailed divergence history of extant right whales, we applied MSMC-IM on pairs of right whale species. To estimate the divergence time of each species pair, we calculated the 50% cumulative migration rate, a way of representing divergence time between populations. Interestingly, we found that the 50% cumulative migration rates in each species pair are nearly identical (around 0.7 million years ago) with overlapping 25% to 75% cumulative migration intervals (Fig. 4b to d and h; supplementary fig. S4, Supplementary Material online). Despite the close divergence time of all species pairs, we found continuous gene flow between the North Pacific right whale and southern right whale that lasted until approximately 200,000 years ago, when gene flow in other species pairs already long ceased (Fig. 4d). The difference in gene flow pattern indicates long-lasting isolation with migration between southern right whales and North Pacific right whales, providing solid evidence that late transequatorial migration happened in the Pacific Ocean. Our results reveal a complex divergence pattern of right whales that cannot be represented by a simple dichotomous phylogeny and confirms that the Pacific Ocean was a passage for transequatorial migration.

### MSMC-IM and *D*-statistics Reveal Evidence of Gene flow From the ECWG Bowhead Whale to North Atlantic Right Whale

We detected signals of archaic introgression in MSMC-IM analyses of right whale species pairs (Fig. 4b and c). Archaic introgression peaks, peaks that predate the main divergence peak, are only present in species pairs that include the North Atlantic right whale, showing that this species is the one that carries genomic material from a divergent species. The archaic lineage that contributed to the introgression diverged from right whales at least 2 to 3 million years ago, based on the position of the peak. We then applied MSMC-IM on the bowhead whale and each of the three right whales. We confirmed that the archaic introgression signal within the North Atlantic right whale indeed came from secondary contact with the bowhead whale (Fig. 4g). This secondary contact started during the mid-Pleistocene transition (Clark et al. 2006) and persisted to the recent past until it mingled with phasing error artifacts. This gene flow is most likely unidirectional since we do not detect any archaic introgression signal in bowhead whales (Fig. 4a). We also used cumulative migration rate to estimate the divergence time between the two genera. Consistent with the position of archaic introgression peak in right whales, the divergence time between right whales and bowhead whale is around 3 million years ago (Fig. 4h; supplementary fig. S5, Supplementary Material online).

To test whether the population structure of bowhead whales affects the introgression with the North Atlantic right whale, we applied the *D*-statistic on the North Atlantic right whale, the ECWG, and the EGSB population of bowhead whales. Our results reveal that the ECWG population, which has a geographical distribution that overlaps with the North Atlantic right whales in the North West Atlantic Ocean, from which the North Atlantic right whale individual was sampled, has strong and significant signals of excessive allele sharing (Table 1). Therefore, we confirmed that the ECWG bowhead whale is the major source of introgression. MSMC-IM does not provide additional insight regarding the excessive allele sharing between bowhead whale and North Pacific right whale. Unlike in the case of bowhead—North Atlantic right whale introgression, we found minimal postdivergence gene flow between the bowhead whale and North Pacific right whale (Fig. 4e). We also did not detect signals from ghost introgression in southern right whales (Fig. 4d). Our analyses provide evidence of unidirectional gene flow from the ECWG bowhead whale to North Atlantic right whale, aligning with the mid-Pleistocene transition. This new information adds more details to the previously predicted introgression.

### Sliding Window Statistics and Enrichment Analyses Show that Interspecific Introgression May Have Phenotypic Effects

To identify putative introgression regions in the North Atlantic right whale, we calculated genome-wide sliding window *fdm* statistics. To evaluate the biological and phenotypical consequences of introgression, we extracted genes within regions with high levels of introgression, and performed Gene Ontology (GO) enrichment analysis. Regions with high levels of introgression are defined as sliding windows with a top 5% *fdm* value. The regions with high levels of introgression from the bowhead whale to the North Atlantic right whale are enriched for the GO terms “olfactory receptor activity” and “intracellular chloride channel activity” (Table 2). Our results show the potential phenotypic effect of interspecific introgression in balaenid whale species.

**Table 2:** GO enrichment analysis of introgression regions in North Atlantic right whale

GO term ID	GO term name	Adjusted *P* value
0004984	Olfactory receptor activity	1.9 × 10^−6^
0061778	Intracellular chloride channel activity	1.6 × 10^−4^
0005229	Intracellular calcium activated chloride channel activity	1.5 × 10^−3^

## Discussion

We reconstructed a detailed history of divergence and gene flow within the baleen whale family Balaenidae encompassing all four extant species. Most phylogenetic studies supported either one of the two competing hypotheses regarding the relationship of modern right whales (*Eubalaena* spp.). The first supports a sibling relationship between North Pacific and southern right whale (Rosenbaum et al. 2000; Gaines et al. 2005; Kaliszewska et al. 2005; Churchill et al. 2011; Buono et al. 2017), while the second suggests a sibling relationship between two northern hemisphere species (McGowen et al. 2009; Steeman et al. 2009; McGowen et al. 2020; Wolf et al. 2023). Some studies, however, have reported alternative findings (Bisconti et al. 2017), including instances of reticulated topology (Wolf et al. 2023). In our study, despite employing whole genomic data, we initially encountered challenges in determining the best dichotomous phylogenetic relationship among the three right whales. We successfully addressed this challenge, however, by treating the right whales as populations and leveraging haplotype information. We demonstrated that the differing topologies among these species may result from lasting gene flow between North Pacific and southern right whales. Therefore, we can confirm that the Pacific Ocean served as a passage for late transequatorial migration, which agrees with both fossil evidence from the Taiwan Strait (Tsai and Chang 2019) and divergence estimation from right whales in the Atlantic Ocean (Crossman et al. 2023). The differences in divergence pattern and introgression with archaic species are likely the factors that have hindered past studies from reaching a conclusion on the two competing topologies, as both conditions violate the basic assumption of most phylogenetic models. Indeed, different algorithms capture different aspects of the data. For instance, the multispecies coalescent models implemented in ASTRAL and SNAPP cluster North Pacific and southern right whales together, possibly because of later gene flow resulting from isolation with migration. In contrast, SVDQuartets, known to be less affected by gene flow from isolation with migration (Long and Kubatko 2018), reported a monophyletic northern group.

We estimated the age of extant *Eubalaena* (0.7 M) and Balaenidae (3 M) from relative cross-coalescence rates between pairs of haplotypes from two populations using the model implemented in MSMC-IM. For this analysis, we used bowhead whale (*B. mysticetus*) generation time recalibrated on late Pleistocene glacial cycles. We first show that despite having a remarkably long lifespan, the generation time of bowhead whales (35 years) is likely similar to that of modern right whales (28 to 35 years) (Taylor et al. 2007). The accuracy of this molecular dating method relies on the assumption that the generation time remains constant throughout evolution. However, fossil evidence supports that gigantism evolved independently in the two extant balaenid genera (Bisconti et al. 2021). As generation time is positively correlated with body size, the common ancestors of extant balaenid whales most likely have shorter generation times. Hence, while the age estimation of *Eubalaena* is not affected since giant body size is a synapomorphy of the extant species, the age of Balaenidae is almost certainly an overestimation. Previous molecular clock-based studies varied in estimating ages of extant *Eubalaena* (0.77 to 4.4 M) and Balaenidae (4.38 to 17.1 M) (Sasaki et al. 2005; McGowen et al. 2009; Slater et al. 2010; Hassanin et al. 2012; Slater et al. 2017; Árnason et al. 2018; McGowen et al. 2020; Wolf et al. 2023). Our results support those estimations that are at the lower end of the spectrum.

The distribution of both extant and extinct species of *Balaena* and *Eubalaena* suggests that the two modern genera likely originated in the Northern Hemisphere (Rooney et al. 2001; Bisconti 2003; Kimura 2009; Churchill et al. 2011). Therefore, the early ancestors of right whales would have needed to cross the equator to reach the Southern Ocean. It has been proposed that climate oscillation during the Pleistocene may have contributed to the antitropical distribution of modern right whales (Davies 1963). Even though our estimated divergence time of *Eubalaena* falls within the Pleistocene, we noticed a deep population structure from the MSMC-IM result. This supports the notion that isolation with migration between southern and North Pacific right whales likely started before the Pleistocene epoch. Consequently, the direct influence of climate oscillations or other geological events on the distribution of *Eubalaena* remains unclear. While there is no evidence of transequatorial movement in modern balaenid whales, rare occurrences of both fossil and stranded pygmy right whales north of the equator have been documented (Tsai et al. 2017; Tsai and Mead 2018). Pygmy right whale is typically distributed in the temperate waters of the Southern Hemisphere. The occurrence of a specialized temperate cetacean on the other side of the equator offers a glimpse into the possible evolutionary history of right whales. It is likely that similar rare movements, facilitated by periods of cold climate, established the transequatorial colonies and provided subsequent gene flow. Over time, genetic differentiation driven by geographical isolation gradually reduced the amount of effective gene flow, ultimately resulting in the three populations/species we see today (van Weelden et al. 2021).

Hybridization in cetaceans has been extensively documented (Miralles et al. 2016; Skovrind et al. 2019; Jossey et al. 2024), yet the extent to which hybrids contribute to gene flow remains debated. Ancient introgressions, which occur at the bases of adaptive radiations, are prevalent across cetacean groups (Árnason et al. 2018; Foote et al. 2019; Moura et al. 2020; Westbury et al. 2023), while more recent interspecific introgressions have also been identified (Westbury et al. 2019; Guo et al. 2021; Jossey et al. 2024). Due to the recent divergence of balaenid whales, we were able to detect not only ancient introgression between balaenid and balaenopterid whales, but also recent introgressions within Balaenidae. We used a combination of likelihood-based, nonparametric, and haplotype-based approaches to detect gene flow among balaenid whales. We characterized both gene flow between sibling taxa (ECWG and EGSB bowhead whale, North Pacific and southern right whale), between more decently related lineages (North Atlantic right whale and bowhead whale), and ancient gene flow between baleen whale families. Historical shifting of distribution in Atlantic bowhead whales due to glacial cycles is an intuitive hypothesis, but can be hard to verify (Foote et al. 2013). MSMC-IM analysis of the ECWG and EGSB populations of bowhead whales was able to provide direct evidence to the hypothesis, showing how informative and powerful a haplotype-based method is. In terms of interspecific introgression, MSMC-IM was able to identify archaic introgression peaks within the North Atlantic right whale, while also providing details on the timing of the event. On the other hand, no archaic introgression peak was found within the bowhead whales, showing that the interspecific introgression between the two species came from the bowhead whale to the North Atlantic right whale. This unidirectional gene flow mirrors a recent report of fin whale to blue whale introgression (Jossey et al. 2024). The biological implication of such phenomenon in cetaceans warrants further research. One shortcoming of MSMC-IM, like all sequential Markovian chain-based methods, is the limited ability to explore the recent past due to phasing error (Wang et al. 2020). In a nonmodel species where the reference panel is unavailable, the only way to overcome the phasing problem is to use accurate long read sequencing data such as PacBio HiFi reads. MSMC-IM also failed to detect introgression signals between North Pacific right whale and bowhead whale, while such signals are strong in *D*-statistics. A potential explanation is that the excessive allele sharing did not originate directly from the introgression between North Pacific right whale and bowhead whale. Instead, it might have resulted from the introgression between southern right whale and a more distantly related unsampled lineage. The deep divergence between the involved lineages, and the potentially ancient origin of the introgression, likely hindered MSMC-IM from detecting the “ghost” introgression in southern right whale.

A limitation of this study is the sample size. Except for the bowhead whale, all conclusions were based on a single diploid individual representing the entire species. However, instead of identifying signatures of recent hybridization, we detected effective gene flow that has been broken down by recombination and has persisted in the population for numerous generations. We argue, therefore, that two haplotypes are sufficient to reliably infer introgression at the population level. Nonetheless, we cannot determine whether the level of introgression varies across different populations within a species, as observed in bowhead whales. Population structure associated with loyalty to breeding waters is known to exist in right whales (Carroll et al. 2020). Additionally, variation of introgression linked to deep population structure was reported in other cetaceans, including orcas, blue whales, and fin whales (Foote et al. 2019; Furni et al. 2024; Jossey et al. 2024). Therefore, to gain a more comprehensive understanding of introgression at a species level, it is essential to include broader sampling from different geographical regions, while testing for hidden population structure for sympatric samples.

Drawing definitive conclusions about adaptive introgression based on only two haplotypes remains challenging. However, regions with high levels of introgression may harbor alleles maintained by natural selection. Adaptive introgression, the process by which gene flow introduces beneficial alleles that enhance fitness in the recipient population, is a plausible explanation in this context. For instance, gene flow from bowhead whales (an Arctic species) to North Atlantic right whales (a temperate species) occurred during the mid-Pleistocene transition, a period of global cooling. It is plausible to speculate that this introgression provided adaptive advantages, potentially facilitating ecological or physiological adjustments to changing environmental conditions. Adaptive introgression has been increasingly documented across a wide range of taxa. The most iconic examples come from our own species, where ancient hybridization events with archaic hominins, such as Neanderthals and Denisovans, have left traces of functional genetic variation (Huerta-Sánchez et al. 2014; Dannemann et al. 2016). Beyond humans, adaptive introgression has been recently reported in several plant and animal lineages, where the genomic basis and molecular mechanisms underlying the associated phenotypes have been largely elucidated (Richards and Martin 2017; Jones et al. 2018; Eberlein et al. 2019).

In our study, GO enrichment analysis suggests that introgression might have influenced olfaction-related genes. Balaenid whales rely on olfaction to sense their prey (Kishida et al. 2015; George and Thewissen 2020), and it is possible that introgressed alleles affect prey identification or prey specificity. However, direct functional validation of these candidate genes is necessary to establish a causal link between introgression and phenotypic adaptation. Testing this hypothesis will require integrating genomic data with ecological observations. Additionally, further exploration of why the X chromosome exhibits an even higher level of introgression than autosomes—a rare phenomenon in mammals (Fraïsse and Sachdeva 2021)—will provide insights into the selection mechanisms and gene flow in these species. Future studies with larger sample sizes, incorporating population-level variation, and combining genomic, ecological, and behavioral data will be critical to clarifying the role of selection in shaping introgression between ecologically distinct species.

In conclusion, our work contributes to enhancing our understanding of the evolution of this ancient and endangered whale family. The findings of this research also have broader implications for related fields in biology, for example, research on aging. As the longest-living mammal, the bowhead whale has been a focus of aging-related studies. Our data underscore the importance of considering interspecific introgression in comparative genomic studies involving bowhead whale and right whales. Furthermore, we discovered that certain gene flow events can be associated with ancient global climate events such as mid-Pleistocene transition and Pleistocene glacial cycles, highlighting how climate changes can profoundly impact cold-adapted species.

## Methods

### Data Collection

We downloaded the reference genomes of the southern right whale, bowhead whale (Keane et al. 2015), blue whale (Bukhman et al. 2024), and pygmy right whale (Wolf et al. 2023). We downloaded whole-genome sequencing short reads from six baleen whale species, including all four extant balaenid and two balaenopterid species, the latter served as outgroups. The balaenid species are North Atlantic right whale, North Pacific right whale, southern right whale, and a bowhead whale from the ECWG population. In addition, we downloaded 12 resequenced bowhead whale samples from the EGSB population (Cerca et al. 2022). The two balaenopterid species were blue whale and minke whale. Detailed information of the samples can be seen in supplementary table S1, Supplementary Material online.

### SNV Calling

Because raw sequencing reads of the pygmy right whale were not available during the time of analysis, we used wgsim v0.3.1-r13 (https://github.com/lh3/wgsim) to simulate 400 million (M) paired short reads from its genome. We aligned short reads of the seven baleen whales to the blue whale genome with bwa mem v0.7.17-r1188 (Li and Durbin 2009) and sorted them with samtools v1.18 (Li et al. 2009). We choose a nonbalaenid species as reference to minimize ascertainment bias. We sampled the mapping files in bam format with samtools so that all balaenid samples matched the sequencing depth of the southern right whale, the species with the lowest sequencing depth (19X). Bcftools v1.18 (Narasimhan et al. 2016) was used to call single nucleotide variations (SNVs) from the aligned sequence files in bam files using default settings. The SNVs were filtered to exclude sites with quality score lower than 20 or a read depth lower than five.

### Phylogenomic Analysis

To estimate the phylogeny of balaenid whales, we used three MCMCs implemented in the programs ASTRAL III v5.7.8 (Zhang et al. 2018), SVDQuartets (Chifman and Kubatko 2014), and SNAPP v1.6.1 (Bryant et al. 2012). The analysis was based on one individual per species and including the ECWG bowhead whale. For the haplotype-based phylogenetic analysis, we first generated consensus autosome genomes for all seven species from the bam files using ANGSD v0.941 (Korneliussen et al. 2014). Second, we used bedtools v2.30.0 (Quinlan 2014) to generate 50 kbp nonoverlapping genomic fragments for each consensus genome. We then apply pargenes v1.2.0 (Morel et al. 2019) on the genomic fragments, which relies on RAXML-NG v1.2.2 (Stamatakis 2014) to estimate individual subtrees from each fragment. Finally, we used ASTRAL III to estimate an overall species tree, where posterior probability was utilized to evaluate the uncertainty in the ASTRAL results. For both SNV-based phylogenetic analyses, we selected biallelic autosomal SNVs and filtered them with vcftools v0.1.17 (Danecek et al. 2011) so that each site was at least 100 bp apart from each other. For SVDQuartets, the Ruby script *convert_vcf_to_nexus.rb* was used to create the input nexus file for the tool implemented in PAUP v4.0a (Swofford and Sullivan 2009). The minke whale, blue whale, and pygmy right whale were set as outgroups and the standard bootstrapping method with 100 replicates was used to run the analysis. For SNAPP, the Ruby script *snapp_prep.rb* was used to prepare the input file with five million MCMC generations. We ran SNAPP implemented in BEAST v2.7.1 (Bouckaert et al. 2019) and used posterior probability to evaluate the uncertainty of the analysis.

### Inferring Introgression with Phylogenetic Network

We used autosomal biallelic SNVs from the same seven baleen whale samples used in the phylogenomic analyses for the phylogenetic network analysis. To minimize the linkage in our SNV data set and for computational efficiency, we sampled SNVs with vcftools so that all remaining SNVs were at least 10 kbp apart. We converted the file format with *vcf2phylip* v2.8 (Ortiz 2019) and used the R package SNP2CF v1.6 (Olave and Meyer 2020) to generate an input CF file for the SNaQ (Solís-Lemus and Ané 2016). SNaQ was run in the Julia v1.8.3 environment using the package PhyloNetworks (Solís-Lemus et al. 2017) to estimate the pseudolikelihoods of networks. Since SNaQ can only infer level one network, we set the number of introgression events from zero to two. The starting topology for SNaQ was the one estimated by SVDQuartets. In cases when the best network reported by SNaQ contains gene flow from a younger lineage to an older lineage, which is temporally impossible to occur, we manually examined all the evaluated networks to find the second-best network. The resulting networks were visualized with the Julia package PhyloPlots. We compared the gene tree discordance that can be explained by dichotomous tree (ILS alone) versus the inferred network with fittedQuartetCF implemented in PhyloNetworks and visualized the result with ggplot2 in the R environment called within Julia.

### Inferring Introgression with *D*-statistics and Sliding Window Statistics

We used Dsuite v0.5r47 (Malinsky et al. 2021) Dtrios to estimate genome-wide *D*-statistics. For introgression among balaenid whales, we used autosomal biallelic SNVs from selected trios of the balaenid species/populations and used the minke whale, blue whale, and pygmy right whale as outgroups. For introgression among major baleen whale lineages, we generated another set of SNVs using the sperm whale genome as a reference. We applied *D*-statistics on the trios of baleen whales: [blue whale, pygmy right whale, southern right whale], using sperm whale as an outgroup. To identify putative introgression regions within the North Atlantic right whale, we estimated genome-wide sliding window fdm statistics with Dinvestigate in the selected set of trios: [North Pacific right whale, North Atlantic right whale, bowhead whale]. Each window is 50 SNVs in size with a step size of 5 SNVs. To infer introgression in the sex chromosome, we extracted SNVs located on the X chromosome of the blue whale. We estimated the level of introgression with SNaQ using the same methods described above.

### Multiple Sequentially Markovian Coalescent and MSMC-IM

To minimize ascertainment bias, we used the bowhead whale genome, sister group to the three right whales among the extant Balaenidae lineages, as the reference for MSMC and MSMC-IM analyses. To identify scaffolds that belong to autosomes and to improve the contiguity of the assembly, we used a chromosome-level assembly of the southern right whale for reference-guided scaffolding of the published bowhead whale reference genome. We aligned the bowhead whale genome to the southern right whale genome using minimap2.1 (Li 2018) and scaffolded the bowhead whale genome into pseudochromosomes with ragtag v2.1.0 (Alonge et al. 2022). We then performed MSMC analysis on each of the four balaenid species (Schiffels and Wang 2020). We separately analyzed the two populations of bowhead whales. For the EGSB bowhead whales, we selected two deeply sequenced individuals as representatives (accession numbers: SRR15669485 and SRR15669487) and aligned the short reads to the improved bowhead whale genome. We generated SNVs-only vcf and bed files of masked regions for each autosome based on sequencing coverage with bcftools and bamCaller.py scripts contained in the MSMC package. We generated inputs for MSMC for each autosome from vcf and masked region bed files using generate_multihetsep.py. We then ran MSMC2 on all autosomes of each species. For MSMC-IM, we first phased the SNVs containing all balaenid whale samples (Browning and Browning 2013). We then used the phased SNVs to estimate cross-coalescence rates for all possible species and population pairs with MSMC2. The MSMC results of the analyzed population/species pairs mentioned above and their cross-coalescence rate were combined with combineCrossCoal.py. We utilized the combined data as input to MSMC-IM (Wang et al. 2020) to estimate migration rate over time in all balaenid species and population pairs. Trio-based estimation of mutation rate was only available in bowhead whale, we therefore used 1.25×10^−8^, the mean mutation rate of studied baleen whales (Suárez-Menéndez et al. 2023), for MSMC-IM. Because phasing error disproportionally affects estimation of both MSMC and MSMC-IM in more recent times, leading to notable artifacts, after examining the outputs of MSMC-IM, we manually masked the four most recent time segments in all results. We plotted the results of MSMC and MSMC-IM with a custom python script. We used the following generation times for each species: (i) North Atlantic right whale: 35.7, (ii) North Pacific right whale: 28, (iii) southern right whale: 29, and (iv) bowhead whale: 35 and 52. Average generation time for a pair of species was calculated using the following equation:


(1) $$G_{12}=2G_{1}G_{2}/(G_{1}+G_{2})$$

where *G*_1_*G*_1_ and *G*_2_*G*_2_ are generation times from each species. We transformed the time unit from generation to year by multiplying generation by average generation time. We used the same methods to perform MSMC-IM analysis between ECWG and EGSB bowhead whales. We calibrated the bowhead whale generation time by manually inspecting the overlapping of migration peaks and glacial periods. We estimated 50% cumulative migration rates in all pairs of right whales, the two bowhead whale populations, and between SRW and ECWG bowhead whales, as an estimation for divergence date. The reason we choose to use SRW to estimate divergence time between BHW is that SRW has no evidence of introgression. The estimation was based on the output of MSCM-IM and calculated using interpolation with a custom python script. When calculating cumulative migration rate, we excluded migration rate estimated in the masked region and the archaic introgression peak region.

### Enrichment Analyses of Genes within Introgression Regions

We identified the sliding windows with top 5% fdm values in the trios [North Pacific right whale, North Atlantic right whale, Bowhead whale]. We then extracted the genes that fall within these regions. We used g:Profiler (Reimand et al. 2007) to perform GO and enrichment analyses on the extracted genes based on the Ensembl annotation of blue whale. We corrected for multitesting using the default g:SCS correction and set the significance cutoff to 0.05.

## Supplementary Material

evaf081_Supplementary_Data

## Data Availability

All sequencing data used in this study came from open databases. Scripts used for the analyses can be found in https://github.com/BaiweiLo/Balaenidae-demography.
