# Supplementary material for: Genome-Wide Demographic Analyses of Balaenid Whales Revealed Complex History of Gene Flow Associated with Past Climate Oscillation
Source: Genome Biol Evol. 2025 May 5;17(5):evaf081. doi: 10.1093/gbe/evaf081 (PMC12082451; doi:10.1093/gbe/evaf081)
Supplement: evaf081_Supplementary_Data [file evaf081_supplementary_data.zip › Supplementary Figures.docx]

**Supplementary Figures**

**Genome-wide demographic analyses of balaenid whales revealed complex history of gene flow associated with past climate oscillation**

Bai-Wei Lo^1,2^, Francisca Martinez-Real^2,3^, Andreas Magg^2,4^, John Pierce Wise Sr^5^, Stefan Mundlos^2,6^, Paolo Franchini^7^

1.Institute for Biology, Freie Universität Berlin, Berlin, Germany

2.Research Group of Development and Disease, Max Planck Institute for Molecular Genetics, Berlin, Germany

3.Andalusian Center for Developmental Biology, CABD (UPO-CSIC-JA), Seville, Spain

4.BCRT, Berlin Institute of Health (BIH), Charité Universitätsmedizin, Berlin, Germany

5.Wise Laboratory of Environmental and Genetic Toxicology, Department of Pharmacology and Toxicology, University of Louisville, Louisville, KY, United States

6.Institute of Medical and Human Genetics, Charité Universitätsmedizin, Berlin, Germany

7.Department of Ecological and Biological Sciences, University of Tuscia, Viale dell'Università s.n.c, Viterbo, Italy

Table of contents

| Figure | page |
| --- | --- |
| Fig. S1. Expected and observed gene tree discordance (quartet CFs) in the balaenid whale phylogenies | 1 |
| Fig. S2. MSMC-IM result of ECWG and EGSB bowhead whales using a generation time of 52 years | 2 |
| Fig. S3. Cumulative migration rate of right whale species pairs estimated from MSMC-IM | 3 |


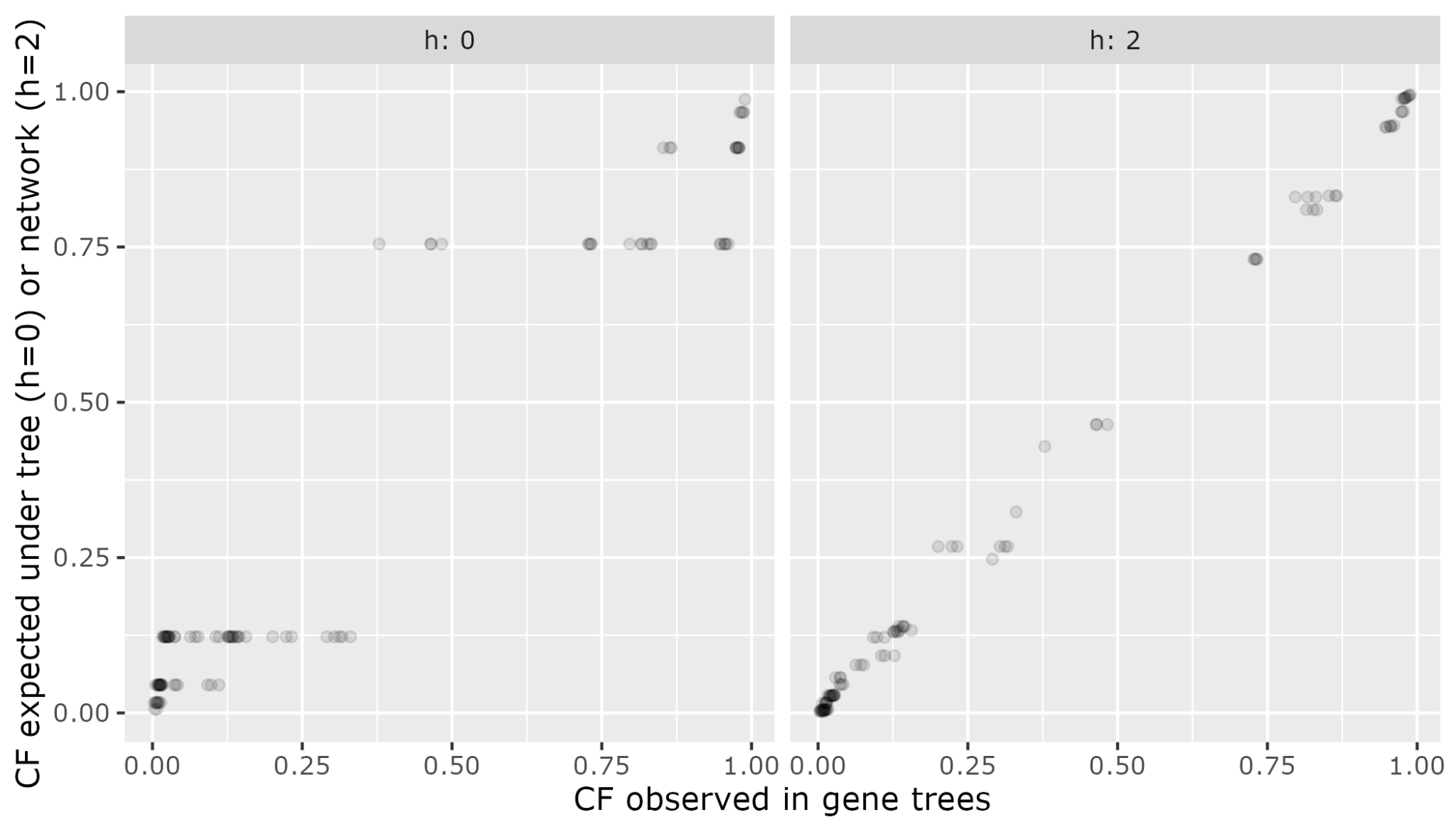


**Supplementary fig. S1.** **Expected and observed gene tree discordance (quartet CFs) in the balaenid whale phylogenies.** A phylogenetic network (right panel) provides a better fitting of data than a phylogenetic tree (left panel). Gene tree discordance can be better explained when gene flow between taxon are allowed, than with incomplete lineage sorting alone.


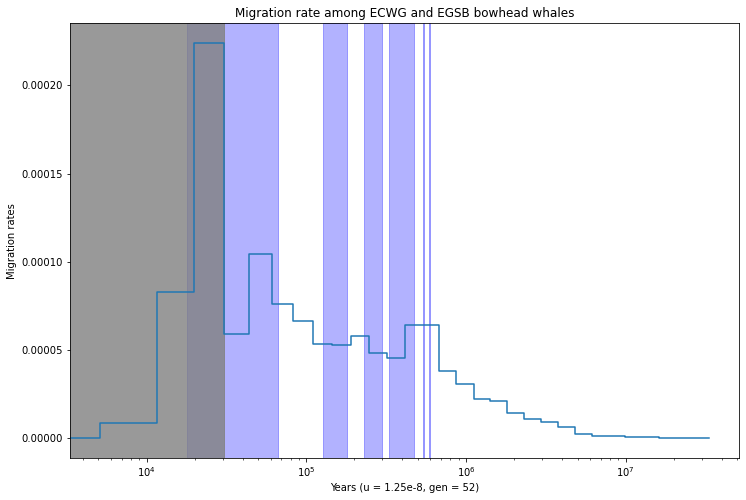


**Supplementary fig. S2. MSMC-IM result of ECWG and EGSB bowhead whales using a generation time of 52 years.**


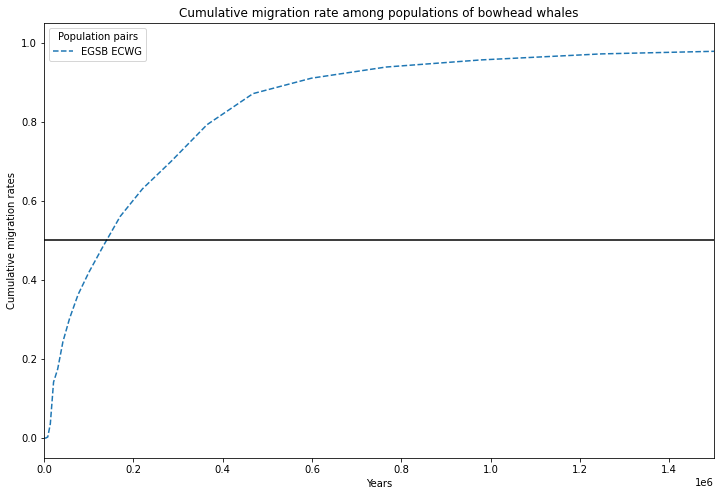


**Supplementary fig. S3. Cumulative migration rate of EGSB and ECWG bowhead whales estimated from MSMC-IM.** The estimate 50% cumulative migration rate between EGSB and ECWG bowhead whales are 139Kya.


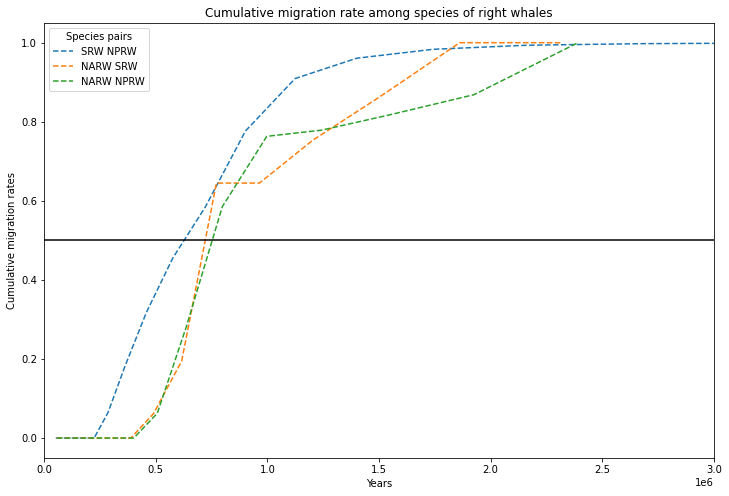


**Supplementary fig. S4. Cumulative migration rate of right whale species pairs estimated from MSMC-IM.** The estimate 50% cumulative migration rate between species pairs are: NPRW/ SRW = 681Kya; NARW/ SRW = 728Kya; NARW/ NPRW = 753Kya.


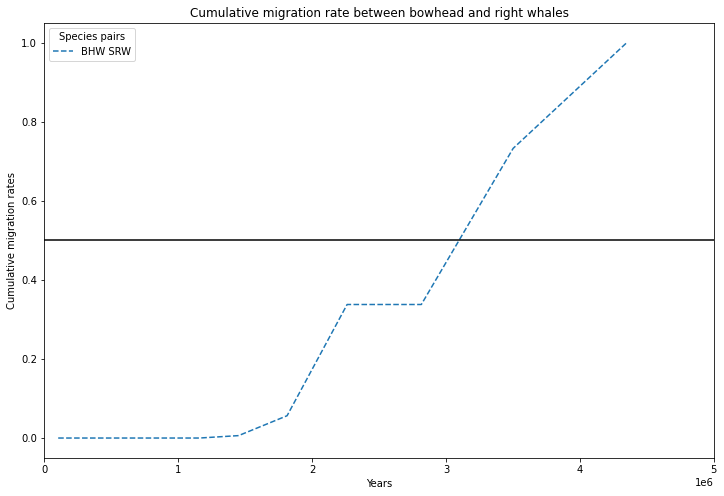


**Supplementary fig. S5. Cumulative migration rate of southern right whale and bowhead whale estimated from MSMC-IM.** The estimate 50% cumulative migration rate across genera is 3.09Mya.
